# Supplementary material for: In Silico Drug Design and Analysis of Dual Amyloid-Beta and Tau Protein-Aggregation Inhibitors for Alzheimer’s Disease Treatment
Source: Molecules. 2023 Feb 1;28(3):1388. doi: 10.3390/molecules28031388 (PMC9919237; doi:10.3390/molecules28031388)
Supplement: Supplementary file 1 [file molecules-28-01388-s001.zip › molecules-2075033-supplementary.pdf]

In silico drug design and analysis of dual Amyloid beta and tau protein aggregation inhibitors for Alzheimer's disease treatment

Nisha Job [1], Venkatesan S. Thimmakondu [2,*], Krishnan Thirumoorthy [1,*]

1 Department of Chemistry, School of Advanced Sciences, Vellore Institute of Technology, Vellore 632 014, Tamil Nadu, India

2 Department of Chemistry and Biochemistry, San Diego State University, San Diego, CA 92182-1030, USA

* Correspondence: thirumoorthy.krishnan@vit.ac.in and vthimmakondusamy@sdsu.edu

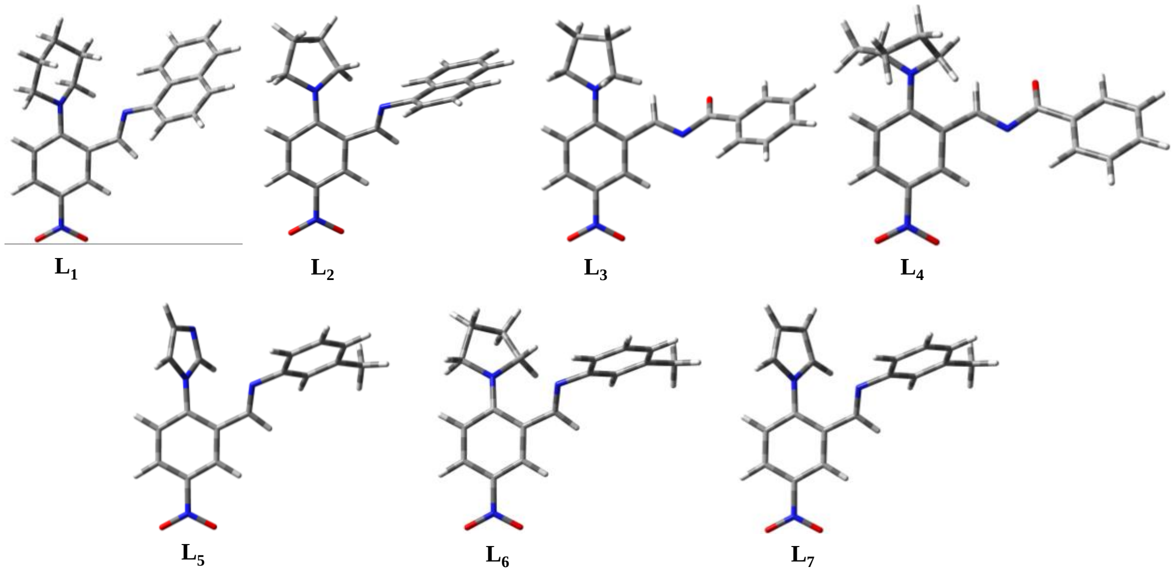

Figure S1. Optimized 3D-tube structures of seven dual inhibitors at ωB97XD/6-311++G(2d,2p) level.

Table S1. Toxicity data of all inhibitor molecules.

Molecule	Toxicity class	Hepatotoxicity	Immunotoxicity	Cytotoxicity
L_1	4	Inactive	Inactive	Inactive
L_2	4	Inactive	Inactive	Inactive
L_3	4	Inactive	Inactive	Inactive
L_4	4	Inactive	Inactive	Inactive
L_5	4	Active	Inactive	Inactive
L_6	4	Inactive	Inactive	Inactive
L_7	4	Active	Inactive	Inactive

Table S2. Type of interactions between selected inhibitor molecules and proteins in the complex.

Molecule	Protein	Interacting Amino acid residue	Distance (Å)	Type of interaction
L_1	Tau protein	LYS-280	4.53	π -alkyl
		LYS-280	5.39	alkyl
		LYS-281	3.64	π-σ
		LYS-281	4.22	π -alkyl
		LEU-282	4.92	alkyl
	Abeta	PHE-8	3.84	π-π stacked
		PHE-8	4.05	π-π stacked
		PHE-8	5.13	π -alkyl
		LYS-16	2.20	Hydrogen bond
		LYS-16	4.63	π-Cation
L_2	tau	ASN-279	3.77	Carbon hydrogen bond
		LYS-280	3.92	π -alkyl
		LYS-281	3.67	π-σ
		LYS-281	4.85	π-alkyl
		LEU-282	5.06	alkyl
	beta	LEU-5	3.97	π-σ
		PHE-8	3.77	π-π stacked
		PHE-8	3.90	π-π stacked
		PHE-8	4.91	π-alkyl
		VAL-12	5.13	alkyl
L_3	tau	ASN-279	2.5	Hydrogen bond
		LYS-280	3.98	π-alkyl
		LYS-281	2.23	Hydrogen bond
		LYS-281	3.83	π-alkyl
	Abeta	PHE-8	3.80	π-π stacked
		PHE-8	5.14	π-alkyl
		ALA-9	4.45	π-alkyl

Table S3. TD-DFT calculations of the excited state of selected inhibitor molecules in the gaseous phase at ωB97XD/6-311++G(2d,2p) level.

Molecule	Wavelength (nm)	Excitation energy (eV)	Oscillator strength, f (a.u)	MO transition	MO Contribution (%)
L_1	291	4.2558	0.3055	H-1→L	26
				H-1→L+1	40
				H→L+1	7
L_2	299	4.1336	0.2468	H-5→L	13
				H-1→L+1	46
				H-1→L+2	13
				H→L	11
L_3	249	4.9696	0.2923	H-4→L+1	22
				H-4→L	10
				H-1→L+2	8

*H-HOMO, L-LUMO

Table S4. TD-DFT calculations of the excited state of selected inhibitor molecules in the solvent (water) phase at ωB97XD/6-311++G(2d,2p) level.

Molecule	Wavelength (nm)	Excitation energy (eV)	Oscillator strength, f (a.u)	MO transition	MO Contribution (%)
L_1	315	3.9272	0.4006	H-3→L+1	6
				H→L	6
				H→L+1	69
L_2	333	3.7137	0.5598	H-1→L	63
				H→L	29
L_3	232	5.3265	0.4720	H-3→L+1	22
				H-2→L+2	29
				H-1→L+2	16

*H-HOMO, L-LUMO

Table S5. The energy of HOMO and LUMO orbitals and their band gap energy of selected inhibitor molecules in the gaseous and solvent phases at ωB97XD/6-311++G(2d,2p) level.

Molecule		E_{HOMO} (eV)	E_{LUMO} (eV)	Energy band gap(eV) ($\Delta E = E_{LUMO} - E_{HOMO}$)
Gaseous phase	L_1	-7.73	-0.74	6.99
	L_2	-7.62	-0.49	7.13
	L_3	-8.43	-0.86	7.57
Solvent phase (water)	L_1	-7.75	-0.95	6.80
	L_2	-7.65	-0.81	6.84
	L_3	-8.16	-0.95	7.21

Table S6. Cartesian coordinates of the optimized geometries of molecules (L_{1-7}) in the gaseous phase at ωB97XD/6-311++G(2d,2p) level.

L_1				L_2			
C	-2.070897	0.903380	0.076644	C	-2.053040	0.726779	0.097792
C	-3.447712	1.186893	0.126575	C	-3.456681	0.751678	0.292012
C	-4.404826	0.212513	-0.047339	C	-4.199572	-0.399414	0.361458
C	-3.998914	-1.092383	-0.270202	C	-3.565724	-1.630871	0.252280
C	-2.660767	-1.422609	-0.275708	C	-2.192387	-1.699004	0.165974
C	-1.676773	-0.455499	-0.089122	C	-1.409940	-0.548736	0.151838
N	-4.998043	-2.137462	-0.470682	N	-4.348379	-2.854705	0.277817
N	-1.143310	1.917252	0.225752	C	0.014483	-0.794752	0.399993
C	-0.233407	2.167084	-0.894511	N	0.800080	0.027840	0.961252
C	1.017537	2.895983	-0.433308	C	2.124079	-0.365283	1.234553
C	0.653150	4.190354	0.287023	C	2.525682	-0.431261	2.544792
C	-0.344239	3.903666	1.404978	C	3.840203	-0.800239	2.879640
C	-1.561233	3.149243	0.880894	C	4.753896	-1.075374	1.903285
C	-0.324101	-0.999921	0.096213	C	4.388835	-0.987319	0.540059
N	0.743908	-0.336791	0.263467	C	3.060186	-0.627024	0.186578
C	1.916871	-1.030091	0.598779	C	5.325113	-1.250001	-0.490764
C	1.962753	-1.876013	1.679199	C	4.974068	-1.150200	-1.805427
C	3.165681	-2.511319	2.047056	C	3.661083	-0.775239	-2.157155
C	4.308161	-2.298789	1.333530	C	2.731896	-0.521650	-1.188839
C	4.301767	-1.431618	0.212760	O	-3.756766	-3.914284	0.161536
C	3.099493	-0.780669	-0.161519	O	-5.555950	-2.753532	0.411616
C	5.471184	-1.195859	-0.549586	H	-3.972585	1.696054	0.349137
C	5.449645	-0.358606	-1.628548	H	-5.272206	-0.359603	0.474752
C	4.251978	0.284968	-2.000562	H	-1.717924	-2.670194	0.165150
C	3.105481	0.076250	-1.286592	H	0.346844	-1.810012	0.156709
O	-4.602488	-3.270298	-0.677749	H	1.807646	-0.201940	3.319901
O	-6.170987	-1.814905	-0.419731	H	4.121767	-0.862600	3.922038
H	-3.772042	2.206297	0.264249	H	5.767152	-1.357180	2.157729
H	-5.456882	0.453266	-0.035318	H	6.333197	-1.530474	-0.213074
H	-2.382898	-2.460297	-0.392745	H	1.732399	-0.231318	-1.475705
H	-0.763632	2.777154	-1.642426	H	5.700842	-1.353251	-2.580149
H	0.022198	1.223431	-1.362268	H	3.389909	-0.687302	-3.200484
H	1.651854	3.101288	-1.297287	N	-1.395414	1.884088	-0.155083

H	1.571068	2.234172	0.234142	C	-2.079006	3.176099	-0.111296
H	0.206938	4.892396	-0.424899	H	-2.464116	3.375346	0.888903
H	1.546177	4.670423	0.688060	H	-2.921205	3.194032	-0.812607
H	-0.678860	4.830195	1.873891	C	-1.009985	4.167539	-0.549629
H	0.133940	3.297323	2.177317	H	-0.373766	4.433203	0.295529
H	-2.121377	3.798420	0.189254	H	-1.440078	5.080564	-0.955060
H	-2.220472	2.897749	1.710211	C	-0.219093	3.356527	-1.573133
H	-0.297918	-2.095330	0.126027	H	0.771099	3.759170	-1.773730
H	1.070877	-2.024581	2.273051	H	-0.765970	3.309523	-2.516548
H	3.174406	-3.166963	2.907170	C	-0.151170	1.978917	-0.922041
H	5.233172	-2.784410	1.615580	H	-0.100983	1.167135	-1.649588
H	6.387661	-1.695155	-0.261947	H	0.710638	1.889963	-0.262486
H	2.187138	0.566157	-1.573160				
H	6.350918	-0.188088	-2.201654				
H	4.242662	0.945441	-2.857089				
L_3				L_4			
C	3.696639	-0.091493	-0.531665	C	-0.984409	1.911264	0.027154
C	4.662272	0.276695	-1.451643	C	-2.110668	2.284946	0.742997
C	2.370378	0.294127	-0.718180	C	0.287389	2.241078	0.499615
C	4.309566	1.034192	-2.562559	C	-1.972720	2.991701	1.934937
C	2.020590	1.054919	-1.831248	C	0.422064	2.951192	1.692895
C	2.990984	1.422974	-2.750982	C	-0.708601	3.324424	2.408088
C	1.358487	-0.137962	0.285011	C	1.469929	1.808104	-0.293162
H	3.949796	-0.678136	0.340297	H	-1.064048	1.362621	-0.903097
H	5.690740	-0.023356	-1.304844	H	-3.096845	2.028362	0.374985
H	5.065547	1.324026	-3.280099	H	-2.853691	3.285523	2.493874
H	2.718760	2.017839	-3.611971	H	-0.603295	3.878362	3.333188
H	0.993432	1.362506	-1.961977	H	1.412203	3.210091	2.043675
O	1.652695	-0.716702	1.305356	O	1.367346	1.243421	-1.360211
C	-4.094814	1.198675	0.219014	C	6.726996	3.274975	0.964765
C	-5.025001	0.172040	0.262465	C	7.744845	2.351359	0.743053
C	-2.741866	0.936423	0.162460	C	5.406544	2.933769	0.767719
C	-4.593057	-1.136163	0.197181	C	7.426417	1.063583	0.374501
C	-2.297184	-0.377679	0.128915	C	5.072432	1.644502	0.356288
C	-3.228338	-1.440972	0.092815	C	6.083651	0.656738	0.213707
C	-0.863683	-0.646038	0.271490	C	3.694001	1.386563	-0.058894
N	0.025135	0.208956	-0.033919	N	2.719785	2.130908	0.299406
H	-6.075916	0.408920	0.328993	H	8.772771	2.652840	0.889032
H	-5.325715	-1.927825	0.204388	H	8.227776	0.349056	0.253438
H	-2.025248	1.743828	0.183451	H	4.624662	3.671557	0.885204
H	-0.588535	-1.612837	0.699514	H	3.522992	0.553562	-0.745219
N	-4.556663	2.586059	0.265004	N	7.067849	4.629132	1.383639
O	-3.718346	3.464532	0.201589	O	6.154834	5.407451	1.586955
O	-5.755208	2.778633	0.360991	O	8.249509	4.899745	1.508311
N	-2.778935	-2.747937	-0.020780	N	5.762461	-0.649067	-0.079790
C	-1.964877	-3.136124	-1.179631	C	6.784490	-1.576772	-0.542402
H	-1.063606	-2.537151	-1.268936	C	4.644791	-1.324579	0.592747
H	-2.548280	-2.999486	-2.099883	H	6.272566	-2.310874	-1.171966
C	-1.675753	-4.617293	-0.931290	H	7.481257	-1.053614	-1.197366
H	-0.712811	-4.736931	-0.438715	C	7.500582	-2.300610	0.616662

H	-1.638058	-5.170772	-1.866977	C	5.120298	-2.588236	1.337306
C	-2.821450	-5.091397	-0.006795	H	4.217059	-0.626513	1.314344
H	-2.425013	-5.422021	0.950986	H	3.857406	-1.582484	-0.123321
H	-3.387958	-5.916953	-0.431965	H	8.419486	-1.782471	0.901437
C	-3.703045	-3.854869	0.184290	H	7.794748	-3.297415	0.275832
H	-4.507564	-3.845841	-0.564855	C	6.556759	-2.406187	1.824651
H	-4.156000	-3.797130	1.172614	H	4.437022	-2.781450	2.166733
				H	5.066041	-3.461724	0.679157
				H	6.618251	-1.490773	2.423007
				H	6.857303	-3.229283	2.475464
L_5				L_6			
C	-2.256323	0.702069	-0.114918	C	-1.483817	1.194323	0.117322
C	-3.638683	0.795001	-0.228518	C	-2.846196	1.399813	0.451288
C	-4.437542	-0.328993	-0.134839	C	-3.753702	0.371130	0.460928
C	-3.823753	-1.553825	0.046719	C	-3.334069	-0.915118	0.145751
C	-2.454160	-1.671743	0.164053	C	-2.000288	-1.171729	-0.088035
C	-1.645041	-0.540668	0.115327	C	-1.050115	-0.157662	-0.044707
N	-4.651793	-2.767255	0.120528	N	-4.295416	-2.002858	0.105430
C	-0.205005	-0.748146	0.374501	C	0.336795	-0.632399	-0.001520
N	0.561979	0.164966	0.792354	N	1.273602	-0.045433	0.622264
O	-4.079495	-3.831462	0.248009	O	-3.890649	-3.113340	-0.194496
O	-5.856371	-2.628012	0.048750	O	-5.457150	-1.744803	0.371125
H	-4.079731	1.763600	-0.415655	H	-3.200406	2.393475	0.671126
H	-5.510955	-0.269617	-0.224786	H	-4.793460	0.552965	0.686329
H	-2.020377	-2.648520	0.322668	H	-1.687406	-2.192894	-0.254711
H	0.142029	-1.778708	0.237284	H	0.500336	-1.610275	-0.469267
N	-1.504909	1.886764	-0.264400	N	-0.674676	2.265650	-0.064550
N	-0.050661	3.335940	-1.084626	C	5.068394	-2.085338	-1.844331
C	-1.625088	3.034117	0.489178	H	5.860968	-2.807790	-1.654194
H	-2.309435	3.098574	1.315653	H	4.335222	-2.550954	-2.502672
C	-0.717968	3.905893	-0.028056	H	5.508824	-1.244733	-2.384315
H	-0.505283	4.909009	0.299002	C	3.150123	-1.082447	-0.564879
C	-0.536654	2.132980	-1.192127	H	2.606606	-1.003137	-1.499112
H	-0.246361	1.391731	-1.918131	C	4.434354	-1.620411	-0.560306
C	4.848926	-1.891341	-0.642578	C	5.121862	-1.689456	0.645731
H	5.686741	-2.394626	-0.161724	H	6.119289	-2.109493	0.669682
H	4.249936	-2.642995	-1.155915	C	4.544432	-1.212741	1.816407
H	5.256893	-1.220100	-1.400784	H	5.093385	-1.268551	2.746895
C	2.680246	-0.870919	0.119119	C	3.275702	-0.662832	1.802051
H	2.222957	-1.232669	-0.794039	H	2.818078	-0.280505	2.703725
C	4.027270	-1.123374	0.357940	C	2.559457	-0.615324	0.607347
C	4.601011	-0.620992	1.521122	C	-1.149777	3.628714	0.169414
H	5.646749	-0.811989	1.725671	H	-1.427816	3.768042	1.214430
C	3.852090	0.138562	2.410363	H	-2.028417	3.843522	-0.449497
H	4.316361	0.532028	3.304481	C	0.021414	4.503568	-0.257465
C	2.519519	0.407505	2.155093	H	0.742031	4.591894	0.556598
H	1.927596	1.013251	2.826437	H	-0.297793	5.503789	-0.541502
C	1.920932	-0.121827	1.015188	C	0.621591	3.701464	-1.409264
				H	1.644203	3.985120	-1.647786
				H	0.014621	3.824290	-2.308132

C	0.533066	2.269221	-0.893679
H	0.443463	1.531016	-1.691821
H	1.404651	2.005972	-0.296034

L7			
C	-1.599926	1.098384	-0.122316
C	-2.959725	1.384249	-0.035337
C	-3.895044	0.375626	0.090976
C	-3.451995	-0.933969	0.096917
C	-2.110044	-1.245065	0.016588
C	-1.157808	-0.234153	-0.058319
N	-4.431909	-2.023517	0.194543
C	0.255851	-0.658466	0.013036
N	1.169834	0.071653	0.490404
O	-4.012141	-3.163896	0.160571
O	-5.602999	-1.717084	0.302677
H	-3.277596	2.414865	-0.100065
H	-4.950819	0.588967	0.152462
H	-1.805866	-2.281450	0.047309
H	0.448117	-1.686560	-0.315631
N	-0.712607	2.174185	-0.302131
C	-0.676053	3.307141	0.478123
H	-1.314473	3.399444	1.339098
C	0.284833	4.136952	-0.021253
H	0.560792	5.094045	0.386061
C	0.222362	2.292859	-1.300649
H	0.349036	1.513977	-2.030968
C	5.014903	-2.158775	-1.719908
H	5.816106	-2.835695	-1.426054
H	4.281850	-2.727620	-2.291230
H	5.443686	-1.408768	-2.387498
C	3.070468	-1.055178	-0.568097
H	2.496063	-1.181508	-1.478067
C	4.386701	-1.503660	-0.518848
C	5.113452	-1.303309	0.649762
H	6.137087	-1.650749	0.708123
C	4.544693	-0.647230	1.733910
H	5.126295	-0.490461	2.632321
C	3.243565	-0.182297	1.670518
H	2.792369	0.345693	2.498598
C	2.490451	-0.409041	0.521410
C	0.849848	3.495084	-1.158001
H	1.626218	3.876770	-1.798028
